# Supplementary material for: Are carbon emissions trading and green financial instruments synergistic? -Comprehensive quantitative research based on content analysis
Source: PLoS One. 2024 Mar 7;19(3):e0298601. doi: 10.1371/journal.pone.0298601 (PMC10919601; doi:10.1371/journal.pone.0298601)
Supplement: S1 Data — (ZIP) [file pone.0298601.s001.zip › Data/地级市Z_F工作报告.zip!/╣π╢1⁄2/╗▌╓▌╩╨/└┤╫╘╣1⁄2╓┌║┼í╛┬φ┐╦╩2╛▌═°í┐_2012.docx]

各位代表：

　　现在，我代表市人民政府向大会报告政府工作，请予审议，并请政协委员和其他列席人员提出意见。

　　过去五年的工作回顾

　　2007年以来，在省委、省政府和市委的正确领导下，在市人大及其常委会和市政协的监督支持下，市政府坚持以邓小平理论、“三个代表”重要思想为指导，深入贯彻落实科学发展观，紧紧围绕建设科学发展惠民之州总目标，大力实施“人本立市、工业富市、文化兴市、创新强市、生态旺市、依法治市”六大战略，全面实施《珠江三角洲地区改革发展规划纲要》（以下简称《珠三角规划纲要》），着力推进“三促进一保持”和“双转移”工作，圆满完成“十一五”规划各项目标任务和市十届政府向全市人民庄严承诺的三件大事，经济社会发展取得重大成就。

　　——— 经济综合实力实现大跨越。预计2011年全市生产总值2060亿元，比上年（下同）增长14.6%，五年（下同）年均增长15%，实现经济总量再造一个惠州的目标。人均生产总值4.4万元，增长10.4%，年均增长10.4%。来源于惠州的财税总收入737.1亿元，增长23.3%，年均增长27.4%；地方财政一般预算收入162.8亿元，增长31.3%，年均增长29.7%，经济总量和财政实力均排名全省第6位。完成固定资产投资1005亿元，增长19.1%，五年累计3733.6亿元，年均增长28.1%；外贸出口224亿美元，增长10.6%，五年累计923.3亿美元，年均增长12.4%；社会消费品零售总额682亿元，增长17%，五年累计2538.5亿元，年均增长18%。五年共引进项目2356宗，投资总额4586亿元，建成中海油1200万吨炼油、95万吨乙烯、TCL液晶模组、科锐LED、抽水蓄能电站、平海电厂一期等一批重大产业项目。成功举办9场 “惠货全国行”展销会，共签订销售合同2472宗，总金额1607.2亿元，履约率达72.3%。工业产品内外销比例由2006年的38.6∶61.4调整为61.6∶38.4。五年新增私营企业1.9万家、个体工商户15.4万户，全年民营经济实现增加值750亿元、税收158亿元，分别是2006年的2.2倍和4.2倍。

　　——— 转变发展方式迈出大步伐。三次产业结构由2006年的7.4∶58∶34.6优化为5.6∶59.6∶34.8。现代农业平稳较快发展，成为全国最大的冬种马铃薯、甜玉米和供港蔬菜生产基地，农业增加值达116亿元，增长4.8%，年均增长4.3%。工业经济迅猛发展，全年实现规模以上工业增加值1010亿元，增长22%，年均增长21.9%。石化产业向中下游发展，形成炼化一体化产业链，67个石化项目落户大亚湾，总投资1553亿元，成为国家级石油化工产业基地。电子信息产业向高端发展，建成液晶电视生产基地和手机生产基地。光电、LED和物联网终端及应用等产业加快发展，成为国家云计算应用创新基地。现代服务业向优质发展，五年引进银行10家、证券保险机构36家，扶持企业上市5家，全市金融机构存款余额突破2300亿元，荣获中国金融生态市称号。新增五星级酒店4家、4A级旅游景区9个，龙门、博罗成为省旅游强县。全年接待旅游总人数2820.9万人次，旅游总收入161亿元，分别增长12.8%和14.4%，年均分别增长15.8%和17.5%。自主创新能力大幅提升，全社会投入研究与开发经费28亿元，占GDP的1.35%，连续四次被评为全国科技进步考核工作先进市，入选省部产学研结合示范市和全省首个技术创新工程试点市。国家石油石化产品质量监督检验中心、国家光电产品光辐射安全监督检验中心落户我市。TCL获评国家数字家庭工程技术研究中心和国家地方联合工程研究中心。五年新增省级以上企业技术研发中心25家、重点实验室3家，认定高新技术企业122家，获得国家专利金奖3项，新增中国驰名商标6件。高新技术产品产值占规模以上工业总产值的比重高达45.6%。

　　——— 城乡面貌发生大变化。完成城乡规划编制和专题研究100多项，中心区控制性详细规划覆盖率85%以上。建成惠南大道、金山大道、惠博大道、合生大桥、西枝江大桥等一大批项目，三环路改造和四环路、惠州大道东段建设加快推进。市区建成区面积达到200平方公里，五年扩大近一倍。建成博物馆、科技馆、文化艺术中心、会展中心、奥林匹克体育场等一大批文体设施，恢复和建成丰渚园、东坡园、合江楼、挂榜阁、叶挺将军纪念园和邓演达纪念园等一批历史建筑和人文景观。建成东江水利枢纽工程，达标加固江海堤围90公里，完成省人大农村机电排灌议案工程221宗、病险水库除险加固工程178宗。完成3360公里通行政村公路和2650公里通自然村村道硬底化改造，90%以上行政村实现“七通八有”。实施西湖美化亮化工程，建成省立绿道网305公里，全市森林覆盖率59.8%，城市人均公共绿地面积15.6平方米，成功创建省林业生态市。五年共否决不符合环保要求项目1070宗，关停所有小火电和立窑小水泥项目，建成污水处理厂24座，淡水河流域整治取得初步成效。通过国家园林城市、国家卫生城市复检，省环保责任考核连续四年被评为优秀。

　　——— 落实《珠三角规划纲要》呈现大成效。以对接珠三角“五个一体化”为突破口，认真落实深莞惠五次联席会议达成的38项合作事项。建成潮莞高速惠州段、惠深沿海高速和广河高速惠州段等3条高速公路，五年新增高速公路里程175.8公里，总里程达453.8公里。莞惠城际轨道、厦深铁路惠州段建设和惠州机场民航复航工作顺利推进。惠州港开通首条国际集装箱航线，新建万吨级深水泊位6个。打通市域边界全部连接路，撤销所有普通公路收费站，率先发行“岭南通·惠州通”公交卡。新增LNG公共汽车200辆，中心城区公交线路进一步优化。大力实施“双转移”战略，龙门、惠东产业转移园累计投入开发建设资金20.3亿元，签订入园项目133个，投资总额228.8亿元，建成投产项目75个。积极参与CEPA和泛珠三角区域经济合作，成为对港澳服务业开放先行先试重点城市。惠台经贸、科技、文化交流不断加强，设立粤台石化合作基地、光电合作基地、科技合作与交流基地。

　　——— 文化建设实现大发展。2008年荣获全国文明城市称号，2011年再次以全国地级市总分第一名的成绩蝉联全国文明城市。建成镇（办）综合文化站73个、行政村（社区）文化室1250个，“农家书屋”实现全覆盖，公共文化场馆免费开放。广播电视“村村通”、公益电影放映、文化信息共享等一批文化惠民工程扎实推进。惠东渔歌、罗浮山百草油制作技艺入选国家级非物质文化遗产，惠阳、龙门、惠东荣获省 “南粤锦绣工程”文化先进县 （区）称号。组建报业传媒集团和广电传媒集团，成立市国有文化资产运营公司，文化体制改革加快推进，被评为全国文化体制改革先进地区。设立文化产业发展专项资金，引导扶持文化产业发展。惠州报业全媒体产业基地、龙门农民画产业园、迪可可儿童城等一批重大文化产业项目顺利推进。文化产业增加值占GDP比重5%以上，高于全省平均水平。成功举办第十三届省运会和第六届省残运会，圆满完成奥运、亚运火炬在惠州传递活动，文明和谐形象得到充分展示。

　　——— 社会事业取得大进步。教育事业蓬勃发展。提前两年通过“普高”验收，提前一年成功创建省教育强市，在全省率先实现城乡教师待遇“两持平一鼓励”。全面实施免费义务教育，完成农村学校危房改造，义务教育规范化学校覆盖率达100%。高中阶段教育毛入学率达92.1%，高考本科上线率达40%。市技师学院建成招生。实施教师免费体检，补贴在职教师攻读硕士、博士。医疗服务体系加快完善。完成73间乡镇卫生院和1052间村卫生站改造，行政村全部实行“一村一站一医生一万元补贴”制度。基层医疗卫生机构综合改革经验在全省推广。建成第一人民医院和第一妇幼保健院。率先开展新生儿疾病免费筛查。连续16年被评为省人口计生工作先进单位，获全国首批人口计生综合改革示范市称号。提前高标准完成援建汶川三江乡任务。基本完成集体林权制度改革。在全国首创“复退军人之家”获中央领导肯定。国防教育、人民防空、民兵预备役工作迈出新步伐，连续四次荣获全国双拥模范城称号，所有建制县（区）荣获省双拥模范县（区）称号。妇女儿童、老年人、残疾人事业取得新发展。外事、侨务、台务、民族、宗教、方志、档案、移民、仲裁、气象、三防、消防、应急、防震、打私等工作取得新进展。

　　——— 民生质量得到大改善。五年累计投入改善民生资金434.4亿元，完成民生工程项目108个。坚持市级新增财力的70%用于民生，民生支出占财政支出的63.9%。累计转移农村富余劳动力19万人、新增城镇就业岗位30万个。城镇居民可支配收入26500元，农民人均纯收入10800元，分别增长12.5%和19%，年均分别增长8.7%和16.2%。在全省率先实行“三网合一”，实现全民医保。75.2万农民参加新农保试点，农村低保和五保供养实现应保尽保。五年建设保障性住房17730套，累计发放住房公积金贷款33.6亿元。稳定物价“三项建设”经验在全国推广，建成100家平价商店，居民消费价格指数为104.9%。累计投入扶贫“双到”资金2.5亿元，帮扶村级扶贫项目349个、贫困户脱贫项目6700多个。74个省级贫困村集体经济收入全部超过5万元，脱贫率达100%，贫困户人均纯收入全部超过2500元，基本实现脱贫目标。完成101.5万平方米农村贫困户危房改造和184宗农村饮水安全工程。深入持久开展社会治安综合治理，荣获全国社会治安综合治理优秀城市。强化安全生产、产品质量和食品药品安全监管，安全生产形势和市场经济秩序进一步好转。连续两年获评中国十佳最具幸福感城市。

　　——— 政府自身建设取得大进展。认真执行市人大及其常委会决议并向其报告工作，自觉接受人民政协民主监督。五年共办理人大代表议案22件、建议618件，政协提案804件、委员意见建议133条，办复率100%。荣获全国“五五”普法先进城市，行政执法责任制示范市建设扎实推进。积极开展审计、监察工作，认真落实政务公开、专家咨询、社会公示和社会听证等制度。完成政府机构“大部制”改革，市政府工作部门由35个精简为32个。行政审批制度改革进一步深化，五年累计缩减行政审批事项320项，在全省率先实现市级行政许可“零收费”。全面实行政府机构编制实名制管理。仲恺高新区完成扩大管理权限改革创新。广泛开展“万众评公务”、“党政领导信箱”、“行风热线”、“惠民在线”等活动。党风廉政建设深入推进，政府效率和服务水平不断提升。

　　过去五年，是建设惠民之州、实现跨越发展的五年，也是人民群众得到实惠最多的五年。五年来的成就，是省委、省政府和市委正确领导的结果，是全市人民共同奋斗的结果，也离不开海内外各界人士的关心支持。在此，我代表市政府，向全市广大工人、农民、知识分子、干部职工，向驻惠人民解放军、武警官兵、人民警察和各民主党派、人民团体、社会各界人士表示崇高的敬意！向长期关心支持我市改革发展的港澳同胞、台湾同胞、海外侨胞和国际友人表示衷心的感谢！

　　在总结成绩的同时，我们也清醒地看到面临的困难和问题：人均发展水平不高，发展质量有待进一步提升；资源环境制约加剧，发展方式有待进一步转变；现代服务业发展缓慢，产业结构有待进一步优化；城乡差距依然较大，统筹发展有待进一步加强；社会建设任务繁重，民生质量有待进一步提高。对此，我们一定要保持清醒认识，增强忧患意识，采取有力措施，认真加以解决。

　　今后五年的目标任务

　　今后五年，是我市加快转变经济发展方式、提升综合竞争力的重要机遇期，也是我市建设科学发展惠民之州的黄金发展期。政府工作的指导思想是：坚持以中国特色社会主义理论体系为指导，以科学发展为主题，以加快转变经济发展方式为主线，以建设科学发展惠民之州为目标，以实施《珠三角规划纲要》为载体，以改善民生为重点，紧紧围绕市第十次党代会提出的 “三件大事”、“八大工程”、“五个发展”的目标任务，努力践行“由民做主”的施政理念，全力推进产业升级，全力推进改革创新，全力推进城乡建设，全力推进文化惠州建设，全力推进各项事业发展，全力推进发展成果共享，实现经济社会发展新跨越，为率先全面建成小康社会和率先基本实现现代化打下坚实基础。

　　——— 全力推进产业升级，经济实力显著增强。高起点谋划、高标准建设“海港”经济，全力打造亿吨大港，力争在“十二五”时期进入全国港口20强。着力发展大亚湾的石化产业和港口物流业、惠阳的现代服务业和先进制造业、惠东的滨海旅游业和清洁能源产业，加速建设 “环大亚湾经济带”。依托惠州机场，加快推进“空港”经济区建设，培育惠州经济发展新引擎。进一步做大做强“电、汽、化”三大先进制造业，大力发展现代服务业，力争到2016年，打造四个产值超千亿元的产业集群，产业规模达1万亿元以上，高新技术产品产值占规模以上工业总产值的比重46%以上，服务业增加值占GDP比重37%以上，全市生产总值3000亿元以上，人均生产总值1万美元以上。

　　——— 全力推进改革创新，发展活力显著提升。坚定不移深化经济、文化、社会改革创新，构建有利于科学发展的体制机制，打造改革的 “惠州模式”。培育扶持和依法管理社会组织，支持、引导其参与社会服务。改革基本公共服务提供方式，引入竞争机制，扩大购买服务，满足群众多样化需求。加大科技创新力度，完善以市场为导向、企业为主体、产学研结合的创新体系，力争到2016年，全社会研究与开发经费支出占GDP比重达2%以上，建成国家级创新平台2个以上、省级创新平台50个以上。

　　——— 全力推进城乡建设，统筹水平显著进步。大力发展县域经济，努力把惠城和惠阳建成现代服务业基地，大亚湾建成世界级石化产业基地和广东重要物流港口基地，仲恺建成国家电子信息产业基地，博罗建成汽车产业基地，惠东建成清洁能源基地和滨海旅游度假基地，龙门建成承接产业转移基地和绿色产业基地。牢固树立“绿色发展、智慧发展、包容发展、人本发展”的理念，建设人民满意城市。强化节能减排和清洁生产，提高资源利用效率。推进水资源“三条红线”管理，加强东江流域水资源保护。始终坚持生态优先，大力推进绿道网和800公里生态景观林带建设，力争森林覆盖率达到62.2%，争创国家生态市，努力争当 “宜居宜业宜游城乡协调发展示范区”。

　　——— 全力推进文化惠州建设，文化软实力显著提高。广泛开展现代公民道德教育，加快构建社会主义核心价值体系。健全文明城市创建长效机制，争当全国文明城市标兵。加快城乡文化基础设施建设，每个镇（办）建成一个综合文化站。加强文化遗产保护与利用，力争成功申报国家级历史文化名城。深入挖掘岭南文化、东江文化和客家文化，积极培育文化创意、文化博览、动漫游戏等新兴产业，巩固壮大广播影视、新闻出版、文艺演出等传统产业，大力培植一批文化企业集团和文化产业园区，力争文化产业增加值占GDP比重达8%，努力把惠州建设成为文化惠民先行区、文化产业新兴区和粤港澳地区重要文化区域。

　　——— 全力推进各项事业发展，社会建设显著加强。大力发展教育事业，实现12年免费教育，加快发展高等教育，力争新增1所本科院校、3所高等职业院校，建成省教育现代化先进市。全面实施“健康惠州”工程，推进卫生事业跨越发展，力争每个县（区）建设1家三甲医院、市区建设3-5家三甲医院。加强民主法制建设，争创全国“六五”普法先进市。创新社会管理理念，加强社区基础建设，探索社会管理“网格化”，推动社会服务精细化。加强社会治安防控、安全生产和食品药品监管，提高社会管理能力和城市管理水平。

　　——— 全力推进发展成果共享，民生质量显著改善。坚持新增财力的70%以上用于民生事业。建成100项群众满意的民生工程，突出解决好就业、教育、医疗和社会保障、收入分配、稳定物价等人民群众最关心、最直接、最现实的利益问题。社会保障体系实现全覆盖，社会保障水平明显提升。推进城乡公共服务一体化，农村基础设施实现“八通八有”。力争完成6.5万套保障性安居工程任务，建立以公共租赁住房为主体的住房保障体系。高标准完成扶贫开发“双到”工作，探索建立扶贫开发长效机制。实现居民收入和经济发展同步增长，城乡居民收入年均增长10%以上，努力让广大人民群众共享更多改革发展的成果。

　　2012年的工作安排

　　2012年是新一届政府开局之年。综合考虑各方面因素，全市经济社会发展的主要预期目标是：生产总值增长11%；人均生产总值增长10%；固定资产投资增长18%；社会消费品零售总额增长15%；外贸出口增长5%；地方财政一般预算收入增长13%；城镇居民人均可支配收入增长10%；农民人均纯收入增长10.5%。居民消费价格指数控制在104%以内；服务业增加值占GDP的36%；城镇登记失业率控制在2.6%以内；户籍人口自然增长率控制在8.6‰以内；节能减排、全社会研究与开发经费支出占GDP比重完成省下达任务。

　　实现上述目标，必须认真贯彻落实中央经济工作会议和省委十届十一次全会精神，结合惠州实际，做到稳中求进、好中求快，在“进”字上做文章，在“快”字上下功夫，突出抓好八项重点工作：

　　一、突出抓好产业结构调整，加快产业转型升级

　　加快发展壮大石化产业。按照“依托大港口，发展大工业，建设大产业”的战略思路，推进中海油二期1000万吨炼油、100万吨乙烯项目开工建设，提升石化产业核心竞争力。促进中海油乐金ABS等项目早日投产，延伸石化产业链。抓好粤台石化合作基地建设，积极引进精细化工项目，加快大亚湾精细化工园和惠阳鸿海精细化工基地建设，形成石化中下游产业群，进一步做大石化产业规模。抓好大亚湾石化区填海扩区工作，拓展产业发展空间。

　　加快发展电子信息产业。实施电子信息产业中长期产业规划，做好产业布局。发挥TCL8.5代液晶面板项目的龙头带动作用，培育发展中下游配套产业，打造全球最具竞争力的液晶电视生产基地。发展移动智能终端产业，打造惠州手机品牌，推动手机产业升级。加快中兴新产业园、雅图视频光电基地等一批项目建设，推动电子信息产业向新型高端发展。

　　加快发展战略性新兴产业。推进省市共建战略性新兴产业基地、粤台光电基地建设。依托科锐LED芯片等龙头项目，积极引进下游及配套企业，打造LED产业基地和LED城市。加快发展云计算智能终端产业集群，壮大移动互联网产业规模，引进培育生物医药、新材料等新兴产业。

　　加快发展汽车产业。加强汽车产业的研究和规划，积极引进有国际品牌、有实力的知名汽车企业，启动博罗、惠阳汽车产业园区建设。依托现有产业基础，承接广州、深圳汽车产业辐射带动，逐步形成以新能源电动汽车带动、汽车零部件产品、汽贸物流维修等相配套的汽车产业发展格局。

　　加快发展清洁能源产业。推进国家石油储备基地、惠东东山海风电场等重点能源项目建设，做好大亚湾LNG电厂二期和国华二期热电联产等项目前期工作，争取将惠州核电项目列入国家能源规划。发展太阳能光伏、动力储能电池等产业。推进天然气管道进入市区和博罗县。

　　加快发展现代服务业。推进荃湾港区国际集装箱码头等项目建设，促进大亚湾、惠阳、惠城现代物流业发展。启动“空港”、“海港”经济区建设。全力推动旅游强县创建工作，加快西湖、罗浮山、南昆山景区和巽寮滨海旅游度假区创建国家5A级景区，推进惠州森林公园等景区和巽寮湾五星级酒店群等项目建设，争取新增4A级景区1个、五星级酒店1家。加快金融业发展，引进金融投资机构和风险投资基金，成立市农村商业银行，发展村镇银行，加强小额贷款和融资担保公司管理，强化金融服务“三农”、中小企业的功能，扶持3家企业上市。培育3G信息、电子商务、会展经济等新兴服务业。

　　二、突出抓好扩大内外需求，确保经济持续稳定增长

　　积极促进投资增长。安排重点建设项目147项，总投资3871亿元，年度计划投资392亿元，其中新开工项目37项，续建项目87项，投产项目23项。深化投融资体制改革，成立和重组若干家投资集团。激活民间投资，鼓励和引导民间资本参与基础设施、市政公用等领域建设。

　　着力推进招商选资。高度重视招商工作，实施项目准入制度，着重引进高端领域、功能契合、产业配套项目。破解资金、土地等要素瓶颈，提高项目落地率并促其尽快产生效益。进一步完善大亚湾开发区、仲恺高新区和产业转移园等园区基础设施建设，增强园区整体招商效应。

　　大力激活消费需求。做好城镇商业布局规划，加快建设高端商贸企业、平价商店、“万村千乡”市场等消费网点，推广连锁经营、网络购物等消费模式，培育旅游、文化、休闲、健康等消费热点，促进消费需求持续快速增长。筹办 “惠货全国行”长沙、哈尔滨大型展销会，创办企业产品常年展销平台，扶持企业开拓内销市场。合理引导住房消费，促进房地产业健康发展。

　　加快发展民营经济。落实促进实体经济加快发展的政策措施，加大扶持力度，切实解决企业发展中遇到的困难。健全中小企业技术创新体系，鼓励和引导民营企业发展新兴产业，推动民营企业转型升级。实施“百十亿”民营企业、民营企业家培育工程，打造100户以上产值超亿元、10户以上产值超10亿元民营企业，培育壮大一批民营龙头企业。

　　不断优化外贸结构。深度开发传统市场，积极拓展新兴市场。筹办1场惠州电子产品国外展销会。提高具有自主品牌、自主知识产权、高附加值产品的出口比重，扩大一般贸易出口和进口规模，积极推进服务贸易，着力发展软件、技术和外包服务。完成100家来料加工企业不停产转型。

　　三、突出抓好《珠三角规划纲要》实施，深化一体化合作

　　加快交通设施无缝连接。编制实施《深莞惠交通运输一体化规划》。积极推进莞惠城际轨道、深惠城际轨道、厦深铁路等跨境交通设施建设。确保仁深高速博深段建成通车，加快广惠高速东延线、惠大高速等项目建设，推进潮莞高速惠东段、仁深高速龙门至博罗段等项目前期工作。加快跨市普通干线公路网建设，完善跨市公交化客运班线，加速融入“珠三角1小时经济生活圈”，主动承接穗深莞辐射带动。

　　加快推进产业融合发展。大力推进龙门、惠东省级产业转移园建设，加快启动“坪清新”片区建设，全面承接产业转移。加强惠州供港农产品生产基地建设，完善供港农产品“绿色通道”。大力引进香港金融服务、港口物流、文化创意、工业设计等现代服务业。

　　加快提升一体化水平。编制实施《深莞惠区域协调发展总体规划》、《城镇群协调发展规划》和《相邻地区基础设施一体化规划》。统筹治理龙岗河-淡水河、石马河潼湖流域跨界污染，关闭流域内20%重污染企业，淡水河、潼湖流域各镇全部建成污水处理设施并投入运营。积极推进与深莞在水利、金融、旅游、科技、公共服务等领域合作。

　　四、突出抓好科技创新，提升经济增长内生动力

　　推进科技创新平台建设。着力抓好西安电子科技大学、武汉大学、石油大学在惠设立国家重点实验室分支机构，组建电子信息研究院，推动石油化工研究院建设，新组建省级以上工程中心2家。加快完善仲恺高新区科技创新服务中心、留学人员创业园等孵化器功能，加快形成孵化集群。加强海洋科技支撑力建设，提高监测水平及风险防范应急能力，实现惠州海洋经济可持续发展。

　　大力发展高新技术产业。加快仲恺高新区国家创新型特色园区和大亚湾科技创新园建设。积极引进高新科技项目，大力发展高新技术企业，力争新认定高新技术企业15家、省级创新型企业3家，新培育民营科技企业20家，高新技术产品产值增长10%以上。

　　深化产学研合作。推进“三部两院一省”产学研合作，加快积聚和整合创新资源，组建产业技术创新联盟。抓好大亚湾开发区和仲恺高新区与高校、科研院所共建产学研创新平台。力争组建产学研技术创新联盟1家、产学研创新平台2家，引进科技特派员50名。支持惠州学院建设研发中心大楼、参与重点产业和重点领域的研发对接与项目合作。

　　推动区域创新体系建设。加快国家知识产权试点城市建设，力争专利申请量、授权量分别增加30%以上。推动深莞惠共同构建开放型区域创新体系，引进深圳股权投资机构在惠建立市级科技创新引导基金。深入开展惠港、惠台科技合作，建设粤台科技合作与交流基地。

　　着力打造人才高地。大力实施“人才引领工程”，着力培养一批先进制造业、现代服务业、现代农业等产业发展的紧缺人才和高素质的现代企业管理人才。以产业和项目为载体，大力引进产业领军人才和科技创新团队。完善人才落户、住房、税收等优惠政策，吸引各类人才扎根惠州。

　　五、突出抓好城乡规划建设，打造宜居宜业宜游城乡

　　优化城乡区域布局。完善惠城、仲恺、惠阳-大亚湾“一城三组团”的城市空间架构。整合惠阳区、惠东县和大亚湾开发区空间资源，发展临海工业、港口物流、滨海旅游产业集群，带动稔平半岛开发，加快建设“环大亚湾经济带”。

　　畅通城市交通网络。启动云山东江大桥、南四环路、惠新大道、金恺路建设和鳄湖路-红花湖路-金榜路路段改造，加快推进惠州大道东段、三环路、惠民大道、惠南大道改造和仲恺大道改扩建等工程，建设三新路口等人行天桥，完善河南岸中心小学周边等道路，全面打通市区的连接路，打造更加通畅的市内交通网络。

　　完善城乡功能配套。加快推进马安围平马围合围加固工程、客运南站综合交通枢纽、莞惠城际轨道西湖站城市综合体、江北市民乐园地下空间综合开发、水东传统风貌街区改造等项目建设。大力推进“三旧”改造，盘活存量土地资源。完善省立绿道配套设施，新建城市和社区绿道网。推进数字城管建设，着力解决城市管理的重点难点问题，不断提升人居环境质量。

　　加强节能减排和环境保护。严格落实节能责任考核，大力推进工业、建筑、商贸、交通、公共机构等领域的节能减排，加快污水处理设施建设，确保完成省下达的任务。加强废气排放监管和重金属污染防治。加快垃圾中转站和生活垃圾无害化处理厂建设，城镇生活垃圾处理率达85%。确保通过国家环保模范城市复查。启动国家生态市创建工作，启动200公里生态景观林带示范段建设，积极推进生态乡镇建设，创建20个生态示范村。

　　六、突出抓好城乡统筹发展，开创“三农”工作新局面

　　加快农业转型升级。加大强农惠农富农政策力度，发展特色效益农业，全市12大特色农产品种植面积210万亩以上。建设现代农业示范基地，新增示范基地20个以上。推进农业产业化经营，新增省级以上农业龙头企业2家、省级农民专业合作社示范社3家。大力推进科技兴农，提升农业技术装备水平，农业科技贡献率达58%。实施品牌发展战略，新增省市名优产品8个以上。拓展农业功能，发展生态观光旅游农业项目。

　　完善农村基础设施。加强水利设施建设和维护，提升防汛抗旱减灾能力，争取博罗成为省农田水利建设示范县。建设整治标准农田12万亩，推进万亩以上灌区改造。稳步推进“村村通自来水”工程，争取惠阳成为省试点区。加快国道205线石坝至新墟段、省道357线平潭至镇隆段、省道244线蓝田至青塘段、省道340线秀埔至石坝段、省道120线潼湖至大岚段、省道255线龙桥大道和龙桥东江大桥改造及县道X213线大埔屯至港口段建设。投资2.4亿元完成800公里新农村公路硬底化改造。投资3.5亿元开展新一轮农村电网建设。深入实施“幸福农村”信息化工程。大力推进名镇名村建设，打造名镇2个、名村20个。

　　巩固提升扶贫成果。推动农业龙头企业与贫困农户结对帮扶，完善“公司+基地+贫困农户”的稳定脱贫致富模式。大力实施扶贫项目，保证贫困村脱贫项目有稳定持续的分红收益、贫困户脱贫项目有稳定的经济效益。加快农村劳动力转移就业，推进扶贫开发“户户有就业”工程。确保扶贫“双到”任务高标准完成。加大帮扶力度，促进革命老区加快发展。

　　七、突出抓好社会事业，促进社会和谐发展

　　加快教育事业发展。进一步巩固“教育创强”成果，实现“镇镇皆强镇”的目标。启动省教育现代化先进市创建工作。实施“人民教育家培养计划”。推进义务教育均衡发展试点工作。加快乡镇规范化中心幼儿园建设。建成市技师学院二期和中职新城。继续支持惠州学院、经济职业技术学院建设。鼓励扶持民办教育，促进教育事业多元化发展。

　　加快文化事业产业发展。加强社会公德、职业道德、家庭美德和个人品德建设，广泛开展群众性精神文明创建活动，推动文明创建向县镇、农村、社区延伸，不断提高城市文明水平，推动文明城市标兵创建工作。创建国家三级以上博物馆3家、国家一级文化馆2家、国家二级以上档案馆2家、省一级以上综合文化站25家。扶持文化精品创作，打造一批特色文化品牌。加强文化遗产保护和修复，积极申报国家历史文化名城。加大监管力度，促进文化市场健康繁荣。探索推动文化与旅游、文化与体育相关产业的融合发展，加快建设惠东动漫产业园、《读者》数字出版等重点文化产业基地。

　　加快卫生等各项事业发展。深化医药卫生体制改革，巩固基层医疗卫生机构综合改革成果，提升公共卫生服务水平。推动第一人民医院创三甲医院。加快发展中医药事业，努力创建中医药强市。广泛开展全民健身活动，推动体育事业加快发展。创新完善计划生育“惠一生”利益导向机制，争创全国计划生育优质服务先进市。积极发展妇女儿童、青少年、老年人、残疾人事业。深入开展“双拥”和优抚安置工作。做好外事、侨务、台务、仲裁、方志、三防、应急、气象、防震、打私、民族宗教、人民防空、民兵预备役等工作。

　　创新和加强社会管理。强化城乡社区自治和服务功能，完善社区约请部门现场办公制度。设立扶持社会组织发展专项资金，培育发展行业协会、公益慈善、科技文化和社区等四类社会组织。创新流动人口服务管理模式，切实维护新生代产业工人合法权益。深化平安惠州建设，进一步推进“东江亮剑”等系列严打整治行动，认真开展打黑除恶专项斗争，依法严厉打击“两抢一盗”等各类违法犯罪，健全社会治安立体防控体系。实行党政领导干部安全生产“一岗双责”，最大限度遏制各类安全事故。整合大亚湾区消防资源，建立和完善统一的应急指挥平台，不断提高应急处置能力。加强食品药品安全监管、价格监督检查，打击欺行霸市、制假售假、商业贿赂，建设社会诚信体系和市场监管体系。加大信访维稳和人民调解工作力度，及时解决群众合理诉求。

　　八、突出抓好十件民生实事，进一步增强人民幸福感

　　始终坚持五个“民共”的核心理念，最大限度实现好、维护好、发展好广大人民群众的根本利益，让人民群众在共建中共享发展成果。市政府向全市人民承诺，今年办好人民群众热切期盼的十件民生实事：

　　一是完成金山河小流域和水环境综合整治。投资5.8亿元，开展河道、河岸以及水环境综合整治，将金山河打造成防洪排涝、休闲观光的城市样板景观带。

　　二是惠州机场实现复航。完成机场跑道改造等配套工程和应急救援等配套设施建设，建成双向8车道机场路，确保惠州机场恢复民航。

　　三是建成6177套保障房和完成4372户农村住房改造。投资9.9亿元，确保金石花园二期等9个保障性住房项目竣工。改造农村困难户住房36万平方米。

　　四是建设100家平价商店和改造5个农贸市场。安排市级价格调节基金2000万元，推进稳定物价“三项建设”，新建100家平价商店。完成市区龙丰、桥东、桥西、下角和下板塘等5个农贸市场改造升级。

　　五是实现全民社保和新增6.5万个就业岗位。6月底前实现城乡居民社会养老保险全覆盖。发行100万张社会保障卡。新增城镇就业岗位6.5万个，解决下岗失业人员再就业2万人，转移农村富余劳动力1.5万人。

　　六是新建东江高级中学。按国家级示范性普通高中标准，新建市东江高级中学，9月建成招生。

　　七是建成2家医院和基本药物制度延伸至行政村。建成市中医医院新院、市中大惠亚医院。全市新增住院病床1500张。所有行政村卫生站全面实施国家基本药物制度。组建统一的120急救指挥体系。

　　八是新建11座污水处理设施和6个休闲公园。加快建设淡水河流域、潼湖流域11座污水处理设施。新建惠州森林公园、金山湖公园一期、望江公园、文星公园、龙丰公园和新湖公园等6个休闲公园。

　　九是推进500个村居文化设施建设。扶持500个行政村（社区）按“五个有”标准建设文化设施。完成10个乡镇、60个自然村体育设施建设。组织农村公益电影放映1.2万场、文艺下乡演出100场。

　　十是新增230辆环保公交车和实行公共自行车租赁服务。新增公交线路15条、环保型公共汽车230辆。建成47.6公里城市绿道，配置1万辆自行车，配套建设100个服务站点，在市区推行公共自行车租赁服务。

　　加强政府自身建设

　　实现未来五年目标，政府责任重大，人民期望殷切。站在新的历史起点上，我们要坚持以人为本，强化由民做主理念，深化改革，开拓创新，锐意进取，努力为建设科学发展惠民之州探新路、谋新篇、开新局。

　　努力建设服务政府。进一步转变政府职能，深化行政审批制度改革，健全县（区）、镇行政服务中心，大力推行电子政务、网上办事，进一步提高政府行政效率和服务水平。加强政务公开工作，深入开展“万众评公务”活动，完善“党政领导信箱”、“行风热线”、“惠民在线”等问政平台，拓展政府与群众沟通的渠道，改进机关作风，全心全意为人民服务。

　　努力建设责任政府。深化行政管理体制和政府机构改革，积极实施富县强镇事权改革，实现管理重心下移。完善责任考核机制，强化领导干部责任意识。严格落实首问责任制、服务承诺制、限时办结制，进一步提升政府执行力和公信力。加大行政问责和责任追究力度，保证政令畅通，推动工作落实。

　　努力建设法治政府。自觉接受人民代表大会及其常委会的法律监督和人民政协的民主监督，认真办理人大代表、政协委员的议案、建议和提案，不断提高办理质量。加大法制宣传教育力度，积极开展“六五”普法。坚持依法行政，全面推进法治城市建设，争创行政执法责任制示范市。

　　努力建设廉洁政府。全面落实党风廉政建设责任制，深入贯彻《廉政准则》。加强监察审计监督，完善对重点项目、重大活动、重要资金使用的专项督查制度，从源头上预防和治理腐败。严肃查处各类违法违纪案件，切实纠正损害群众利益的不正之风，打造清正廉洁政府。

　　各位代表！回首辉煌过去，任何艰难险阻都改变不了惠州人民创造幸福生活的愿望；展望美好未来，任何困难挑战都阻挡不了惠州人民昂首前进的步伐。让我们更加紧密地团结在以胡锦涛同志为总书记的党中央周围，深入贯彻落实科学发展观，在省委、省政府和市委的正确领导下，同心协力，奋发进取，推动惠州经济社会又好又快发展，为加快转型升级、谱写科学发展惠民之州新篇章而努力奋斗！
